# Healthy aging—would cytotoxic T lymphocytes stand out?

**DOI:** 10.1038/s41420-023-01699-1

**Published:** 2023-10-24

**Authors:** Tianyu Zhang, Jinjin Zhang, Shixuan Wang

**Affiliations:** 1 National Clinical Research Center for Obstetrical and Gynecological Diseases, Tongji Hospital, Tongji Medical College, Huazhong University of Science and Technology, Wuhan, China; 2 Key Laboratory of Cancer Invasion and Metastasis, Ministry of Education, Tongji Hospital, Tongji Medical College, Huazhong University of Science and Technology, Wuhan, China; 3 Department of Obstetrics and Gynecology, Tongji Hospital, Tongji Medical College, Huazhong University of Science and Technology, Wuhan, China

**Keywords:** Immunology, Medical research

## Abstract

Senescent cells (SnCs) accumulate in multiple tissue types with the process of individual aging, hindering tissue renewal and driving multiple aging-related disorders. Aside from senescence-associated secretory phenotype (SASP) neutralization and senolytics, there is an emerging senolysis strategy to eliminate SnCs by mobilizing the immunosurveillance function of cytotoxic T lymphocytes (CTLs).

## Emerging interest in senolysis during healthy aging

Regulated by intricate internal and external factors including genomic features, neuroendocrine, lifestyle choices, and medical interventions, the aging process contains structural and functional changes in multiple systems. The chronic pro-inflammatory microenvironment and cellular damage accelerate the cellular senescent process which generates SnCs. With their continuous production of SASP, SnCs hinder tissue renewal and accelerate pathological deterioration, thus damaging the development and maintenance of functional ability, which is the major concern of healthy aging. Suppression and neutralization of SASP have been effective to alleviate aging. Unfortunately, these methods require continuous dosing which may bring about side effects. Worse still, they are not capable of eliminating SnCs directly and cutting off the production of SASP from the source [1].

An interesting study demonstrated that a small number of SnCs transplanted into young mice were sufficient to cause persistent physical dysfunction. Even worse, the transplanted SnCs could stimulate cellular senescence in the young host. However, the removal of SnCs (senolysis) with senolytics improved the health condition in old mice and reduced the tumorigenic potential of SnCs [2]. This study provides the groundbreaking proof-of-concept for senolysis therapy targeted on SnCs to benefit healthy aging [3].

**Fig. 1:**
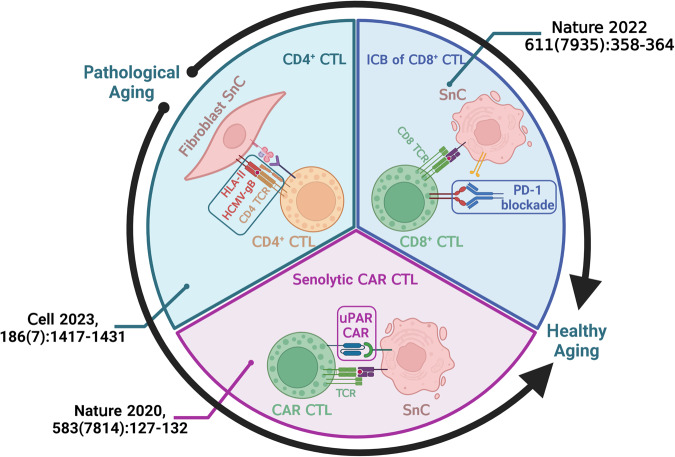
Overview of mobilizing CTLs as a senolysis strategy. Upper left: Targeted on the HCMV-gB (human cytomegalovirus glycoprotein B) antigen presented by HLA-II, the natural clearance of SnCs in women’s skin tissue is carried out prominently by CD4+ CTLs. Upper right: Immune checkpoint blockade (ICB) of PD-1 on CD8+ CTLs lowers the chance of immune evasion of SnCs in mice and human fibroblast cell line (hHCA2). Bottom: Artificially modified CAR T cells with senolysis function in mice. Created with BioRender.com.

The immune system primarily takes the responsibility of identifying and recycling irregular cells, thus ensuring an endogenous approach to achieve healthy aging. However, the immunosurveillance function gradually loses its balance with cellular and microenvironmental changes in the aging process, indulging the irreversible aging process. Hence, the restoration and mobilization of immunosurveillance could be an entry point to boost healthy aging (Fig. 1).

## The potential of using CTLs as a senolysis strategy

CTLs, as an important part of the immune system, mainly include CD4^+^ CTLs, CD8^+^ CTLs, and artificially modified cytotoxic chimeric antigen receptor T cells (CAR T cells). CTLs bind with antigen-presenting target cells, releasing perforin and granzyme which induce the death of target cells [4]. Studies have suggested the well-established role of CTLs in eliminating cancer cells selectively [5]. Could this theory be applied to the elimination of SnCs effectively? p21, a classical marker of cellular senescence, was found to induce CXCL14 and other immune-modulatory factors expression in hepatocytes. These immune modulatory factors further boosted M1 macrophage differentiation and the recruitment of CTLs to p21-expressing hepatocytes, strongly suggesting that CTLs may contribute to the immune clearance of SnCs [6].

## CD4^+^ CTLs

Previously, a detailed landscape analysis of CD4^+^ T cell subset reorganization in the spleen and blood of young and old mice demonstrated that CD4^+^ CTLs accumulate significantly during aging [7]. In tune with this, it was also revealed that CD4^+^ CTLs are markedly elevated in the peripheral blood mononuclear cells of supercentenarians [8]. Despite the lack of direct evidence, these studies suggest that CD4^+^ CTLs may take part in the postponement of the aging process.

A recent study showed a significant increase of senescent fibroblasts in the skin of old women compared to that of young women, with the amount of senescent fibroblast positively correlated with the CD4^+^ CTLs in the skin [9]. The CD4^+^ CTLs could enter the skin tissue by strong CXCL9 chemotaxis expression in human skin and remove the senescent fibroblasts by recognizing the HCMV-gB antigen presented by HLA-II on the senescent fibroblasts, indicating that CD4^+^ CTLs may be an independent manipulator of SnCs in the skin. These studies suggested that an appropriate overexpression of CXCL9 in skin tissues to recruit CD4^+^ CTLs or the manufacture of HCMV-gB antigen vaccines and topical drugs could be promising strategies to alleviate the aging of the skin.

## CD8^+^ CTLs

CD8^+^ CTLs, along with immune checkpoint blockade (ICB), have also been drawing increasing attention in exploring the clearance strategies of SnCs. One study found that blockade of programmed cell death protein 1 (PD-1) could ameliorate the aging phenotype of kidneys in aged mice, yet the underlying mechanism was unclear [10]. Another report clearly demonstrated that certain SnCs subsets can escape CD8^+^ CTL immunosurveillance through their heterogeneous expression of PD-L1 [11]. The authors first identified that PD-L1^+^p16^high^ SnCs accumulate in multiple organs of mice and human fibroblast cell lines (hHCA2) during aging. Further, senescence-induced phenotypes were more positively correlated with the presence and selective accumulation of PD-L1^+^p16^high^ SnCs rather than PD-L1^-^p16^high^ SnCs, suggesting that there is still heterogeneity within the SnCs groups. It is worth noting that anti-PD-1 antibodies not only elevated the cytotoxicity of CD8^+^ CTLs but also improved the phenotype of multiple organs. These findings provide the possibility for the application of CD8^+^ CTLs and ICB in senolysis therapy. Besides PD-1, other immune checkpoint targets should be verified to optimize senolysis against aging-related disorders.

## CAR T lymphocytes

CAR T cells, targeted on antigens of cancer cells, have been widely used in the treatment of certain cancers. Notably, CAR T cells were repurposed for senolysis in recent studies. Urokinase-type plasminogen activator receptor (uPAR) is one of the specifically upregulated transmembrane receptors on senescent cells and its soluble form consists of a part of SASP. An interesting uPAR-specific cytotoxic CAR T cell was engineered [12]. In CCl4 and NASH-induced liver fibrosis models in mice, CAR T cells effectively reduced fibrosis and senescent cells in the liver and significantly improved liver function in an efficient and safe manner. Another study engineered CAR T cells targeted natural killer group 2 member D ligands (NKG2DLs), a newly identified senescent-specific immunogen. These CAR T cells successfully eliminated the senescent human cells induced in vitro and slackened pathological deteriorations in aged mice safely [13]. Based on the differences in senescent profiles among systems, CAR T cells engineered to act on SnCs of a specific system or organ could be an enticing project in the future.

## Interrelationship and distinctions among the CTLs in senolysis

While CD8^+^ CTLs are recognized as the dominant CTL group eliminating SnCs in most tissue or organ types, CD4^+^ CTLs usually stay as a small subset in the same areas. However, the amount of CD4^+^ CTLs subset can increase particularly in aging circumstances with long-term and repeated exposure to chronic viral infection [14]. Since chronic HCMV infection is commonly seen in the aging skin tissue and viral antigen presentation is carried out by HLA-II class, CD4^+^ CTLs become the prominent CTLs in eliminating SnCs of the aging human skin [9]. Hence, the CTL subset composition could be different across systems based on their senescence-specific immunogen feature. While the immunologic recognition of SnCs by CD4^+^ CTLs and CD8^+^ CTLs are still restricted by HLA presentation and the low expression of certain senescent-specific immunogens, artificially engineered CARs offer T cells the ability to target senescent-specific antigens which are neglected by immunosurveillance. With the expanding discovery of senescent markers, a series of CAR T cells targeted on different antigens could be applied to tackle the situation of antigen loss in long-term treatments [15].

## Concluding remarks

Together, these results highlight the potential application of CTLs for senolysis and healthy aging. Immunosurveillance on aging is a complex yet poorly understood natural process. Revealing attempts have been made to identify prominent senescent hallmarks that activate CTLs. Furthermore, the demonstration of how SnCs are eliminated by CTLs in natural processes offers potential interference approaches. The exploratory application of ICB and CAR T cells provides strategies to prevent SnCs from escaping immunosurveillance. With the discovery of new aging hallmarks and successful mobilization of CTLs, future senolysis may contribute to healthy aging and prevent aging-related diseases.

## References

[1] Baker DJ, Childs BG, Durik M, Wijers ME, Sieben CJ, Zhong J (2016). Naturally occurring p16(Ink4a)-positive cells shorten healthy lifespan. Nature.

[2] Xu M, Pirtskhalava T, Farr JN, Weigand BM, Palmer AK, Weivoda MM (2018). Senolytics improve physical function and increase lifespan in old age. Nat Med.

[3] Childs BG, Gluscevic M, Baker DJ, Laberge RM, Marquess D, Dananberg J (2017). Senescent cells: an emerging target for diseases of ageing. Nat Rev Drug Discov.

[4] Golstein P, Griffiths GM (2018). An early history of T cell-mediated cytotoxicity. Nat Rev Immunol.

[5] Waldman AD, Fritz JM, Lenardo MJ (2020). A guide to cancer immunotherapy: from T cell basic science to clinical practice. Nat Rev Immunol.

[6] Sturmlechner I, Zhang C, Sine CC, van Deursen EJ, Jeganathan KB, Hamada N (2021). p21 produces a bioactive secretome that places stressed cells under immunosurveillance. Science (New York, NY).

[7] Elyahu Y, Hekselman I, Eizenberg-Magar I, Berner O, Strominger I, Schiller M (2019). Aging promotes reorganization of the CD4 T cell landscape toward extreme regulatory and effector phenotypes. Sci Adv.

[8] Hashimoto K, Kouno T, Ikawa T, Hayatsu N, Miyajima Y, Yabukami H (2019). Single-cell transcriptomics reveals expansion of cytotoxic CD4 T cells in supercentenarians. Proc Natl Acad Sci USA.

[9] Hasegawa T, Oka T, Son HG, Oliver-García VS, Azin M, Eisenhaure TM (2023). Cytotoxic CD4(+) T cells eliminate senescent cells by targeting cytomegalovirus antigen. Cell.

[10] Pippin JW, Kaverina N, Wang Y, Eng DG, Zeng Y, Tran U (2022). Upregulated PD-1 signaling antagonizes glomerular health in aged kidneys and disease. J Clin Investig.

[11] Wang TW, Johmura Y, Suzuki N, Omori S, Migita T, Yamaguchi K (2022). Blocking PD-L1-PD-1 improves senescence surveillance and ageing phenotypes. Nature.

[12] Amor C, Feucht J, Leibold J, Ho YJ, Zhu C, Alonso-Curbelo D (2020). Senolytic CAR T cells reverse senescence-associated pathologies. Nature.

[13] Yang D, Sun B, Li S, Wei W, Liu X, Cui X (2023). NKG2D-CAR T cells eliminate senescent cells in aged mice and nonhuman primates. Sci Transl Med.

[14] Cheroutre H, Husain MM (2013). CD4 CTL: living up to the challenge. Semin Immunol.

[15] Larson RC, Maus MV (2021). Recent advances and discoveries in the mechanisms and functions of CAR T cells. Nat Rev Cancer.

